# An Automated Quiet Sleep Detection Approach in Preterm Infants as a Gateway to Assess Brain Maturation

**DOI:** 10.1142/S012906571750023X

**Published:** 2017-02-24

**Authors:** Anneleen Dereymaeker, Kirubin Pillay, Jan Vervisch, Sabine Van Huffel, Gunnar Naulaers, Katrien Jansen, Maarten De Vos

**Affiliations:** Department of Development and Regeneration, University Hospitals Leuven, Neonatal Intensive Care Unit, KU Leuven (University of Leuven), Leuven, Belgium; Institute of Biomedical Engineering (IBME), Department of Engineering Science, University of Oxford, Oxford, United Kingdom; Department of Development and Regeneration, University Hospitals Leuven, Neonatal Intensive Care, Unit & Child Neurology, KU Leuven, (University of Leuven), Leuven, Belgium; Department of Electrical Engineering-ESAT, Division Stadius, KU Leuven (University of Leuven), Leuven, Belgium and imec, Leuven, Belgium; Department of Development and Regeneration, University Hospitals Leuven, Neonatal Intensive Care Unit, KU Leuven (University of Leuven), Leuven, Belgium; Department of Development and Regeneration, University Hospitals Leuven, Neonatal Intensive Care Unit & Child Neurology, KU Leuven (University of Leuven), Leuven, Belgium; Institute of Biomedical Engineering (IBME), Department of Engineering Science, University of Oxford, Old Road Campus Research Building, OX3 7DG, Oxford, United Kingdom

**Keywords:** EEG, preterm neonate, quiet sleep, CLASS, automated sleep detection, brain maturation

## Abstract

Sleep state development in preterm neonates can provide crucial information regarding functional brain maturation and give insight into neurological well being. However, visual labeling of sleep stages from EEG requires expertise and is very time consuming, prompting the need for an automated procedure. We present a robust method for automated detection of preterm sleep from EEG, over a wide postmenstrual age (PMA = gestational age + postnatal age) range, focusing first on Quiet Sleep (QS) as an initial marker for sleep assessment. Our algorithm, CLuster-based Adaptive Sleep Staging (CLASS), detects QS if it remains relatively more discontinuous than non-QS over PMA. CLASS was optimized on a training set of 34 recordings aged 27–42 weeks PMA, and performance then assessed on a distinct test set of 55 recordings of the same age range. Results were compared to visual QS labeling from two independent raters (with inter-rater agreement Kappa = 0. 93), using Sensitivity, Specificity, Detection Factor (DF = proportion of visual QS periods correctly detected by CLASS) and Misclassification Factor (MF = proportion of CLASS-detected QS periods that are misclassified). CLASS performance proved optimal across recordings at 31–38 weeks (median DF = 1.0, median MF 0–0.25, median Sensitivity 0.93–1.0, and median Specificity 0.80–0.91 across this age range), with minimal misclassifications at 35–36 weeks (median MF = 0). To illustrate the potential of CLASS in facilitating clinical research, normal maturational trends over PMA were derived from CLASS-estimated QS periods, visual QS estimates, and nonstate specific periods (containing QS and non-QS) in the EEG recording. CLASS QS trends agreed with those from visual QS, with both showing stronger correlations than nonstate specific trends. This highlights the benefit of automated QS detection for exploring brain maturation.

## 1 Introduction

Despite advances in perinatal and neonatal intensive care, preterm birth is still associated with a high risk of neurological disabilities that will manifest later in life.^1^–^4^ Intensive monitoring of these vulnerable preterm infants is increasingly complemented with bedside neuromonitoring to achieve optimal insight into neurological well being. Assessment of neurological function by electroencephalogram (EEG) in this intensive period of neonatal care, can help to identify the influence of various endogenous and exogenous disturbances on the maturation of cortical activity,^5^–^7^ with the ultimate goal to improve therapeutic strategies and neurodevelopmental outcome. Previous research has highlighted sleep ontogenesis (the changing nature of sleep states with age) as an important neurophysiological biomarker of functional brain development, based on the visual labeling of sleep states by expert clinicians using full polysomnography (PSG) traces.^8^–^11^ This highlights the importance to support and optimize neonatal sleep in the Neonatal Intensive Care Units (NICUs).

A significant organization of these sleep states occurs from 28 and 29 weeks of gestational age. Deeper brain nuclei modulate the first reflections of sleep states in the cortical activity and the differentiation between Active Sleep (AS, also known as Rapid-Eye Movement (REM) sleep) and Quiet Sleep (QS, also known as non-REM (NREM) sleep) from EEG can be made.^11^,^12^ As more complex sleep states follow the growth of major cortical afferent connections,^13^ the organization of the four traditional sleep states and wakefulness are established near term age of 36–40 weeks postmenstrual age (PMA = gestational age + postnatal age).^8^,^11^,^12^

In order to expand existing knowledge of extrauterine brain development and to translate these neurophysiologic findings to clinical practice, an automated approach to detect preterm sleep states is necessary, as visual labeling of sleep by clinicians requires particular expertise^12^ and is very time consuming. This can potentially open the possibility for scoring sleep in real time, useful in the day-to-day monitoring of preterms, for assessing optimal periods for feeding and perinatal care. Producing a method for automated and robust detection of preterm sleep states would also allow a faster and more efficient collection of sleep-labeled recordings, from which, one can define objective quantitative maturational characteristics of cortical function for the definition of normal maturational trends, with the ultimate aim to detect abnormal patterns in preterm brain maturation (dysmaturity).^5^,^9^,^14^–^18^

This motivates our choice to develop an automated algorithm for sleep scoring, focusing first on QS as an initial primary marker for sleep assessment. Some EEG background abnormalities are only apparent in QS making EEG more discontinuous and asynchronous, reflecting more subtle alterations in brain function.^9^,^14^,^15^,^19^,^20^ Furthermore, QS contains relatively low levels of artifacts (due to very little motion of the preterm during this state), potentially allowing for a more robust calculation of maturational trends from QS and an automation of the full procedure, from QS detection to dysmaturity assessment.

Current methods for automated QS detection in preterm infants are limited, however.^21^,^22^ Turnbull *et al.* focused on detecting a particular discontinuous EEG pattern, known as *tracé alternant*, to subsequently classify these periods as QS.^23^ While proving reliable for *tracé alternant* detection, this was not sufficient to infer QS over a wide age range, as *tracé alternant* is only present at term age and does not define the entirety of QS at this age (e.g. there is also the presence of high voltage slow wave QS). Palmu *et al.* developed an algorithm based on detecting the percentage of burst periods in the EEG, defined as spontaneous activity transients.^24^ Regions with the lowest percentage of spontaneous activity transients (SAT%) over time were observed in the deeper periods of sleep, often corresponding to rudimentary QS.^22^ However, the SAT% method has only been performed on specifically selected clean EEG recordings for ages <32 weeks PMA and has not yet been used to explicitly detect QS.

There remains no quantitative method to detect QS robustly in the vulnerable preterm age range >32 weeks PMA. Krajca *et al.* proposed a method that involved segmenting the EEG periods and extracting simple time-domain and frequency-domain features which were then clustered into distinct groups. The evolution of these cluster labels over time reflected transitions into and out of QS.^25^–^27^ However, the method was vulnerable to high power artifacts and the concept was illustrated only on a single recording at term age.

In this study, our aim is to build on this approach and develop an automated QS detection algorithm that performs robustly over a wide PMA range and stage of brain development, to be directly applicable for the clinical setting. We present a novel method, called CLuster-based Adaptive Sleep Staging (CLASS), with the performance of CLASS QS detections compared to the clinicians’ visual labeling of QS. We also illustrate from these results, the potential of CLASS QS estimates for defining normal maturational trends, and compare this to the trends derived from the visual labeling of QS, as well as from nonstate specific EEG epochs (containing QS and non-QS). This is to assess if normal maturational trends are improved when focusing on QS specifically, and if it can then be defined using CLASS in a fully automated approach.

## 2 Methods

### 2.1 Data acquisition and EEG recordings

This study was performed at the NICU of the University Hospitals of Leuven, Belgium and approved by the Ethics Committee of the University Hospitals of Leuven, Belgium. Neonates were enrolled in the study after informed parental consent. The dataset consisted of 26 preterm neonates with gestational age ≤32 weeks. Neonates were retrospectively selected as ‘normal’, based on strict inclusion criteria: (1) A normal neurodevelopmental outcome score at 9 and 24 months corrected age (Bayley Scales of Infant Development-II, mental and motor function >85), (2) no use of any sedative or anti-epileptic medication during EEG registration, and (3) the absence of a severe cerebral lesion (normal cerebral ultrasonography or intraventricular hemorrhage grade ≤ II, no periventricular leukomalacia or ventricular dilatation >p97).

**Fig. 1:**
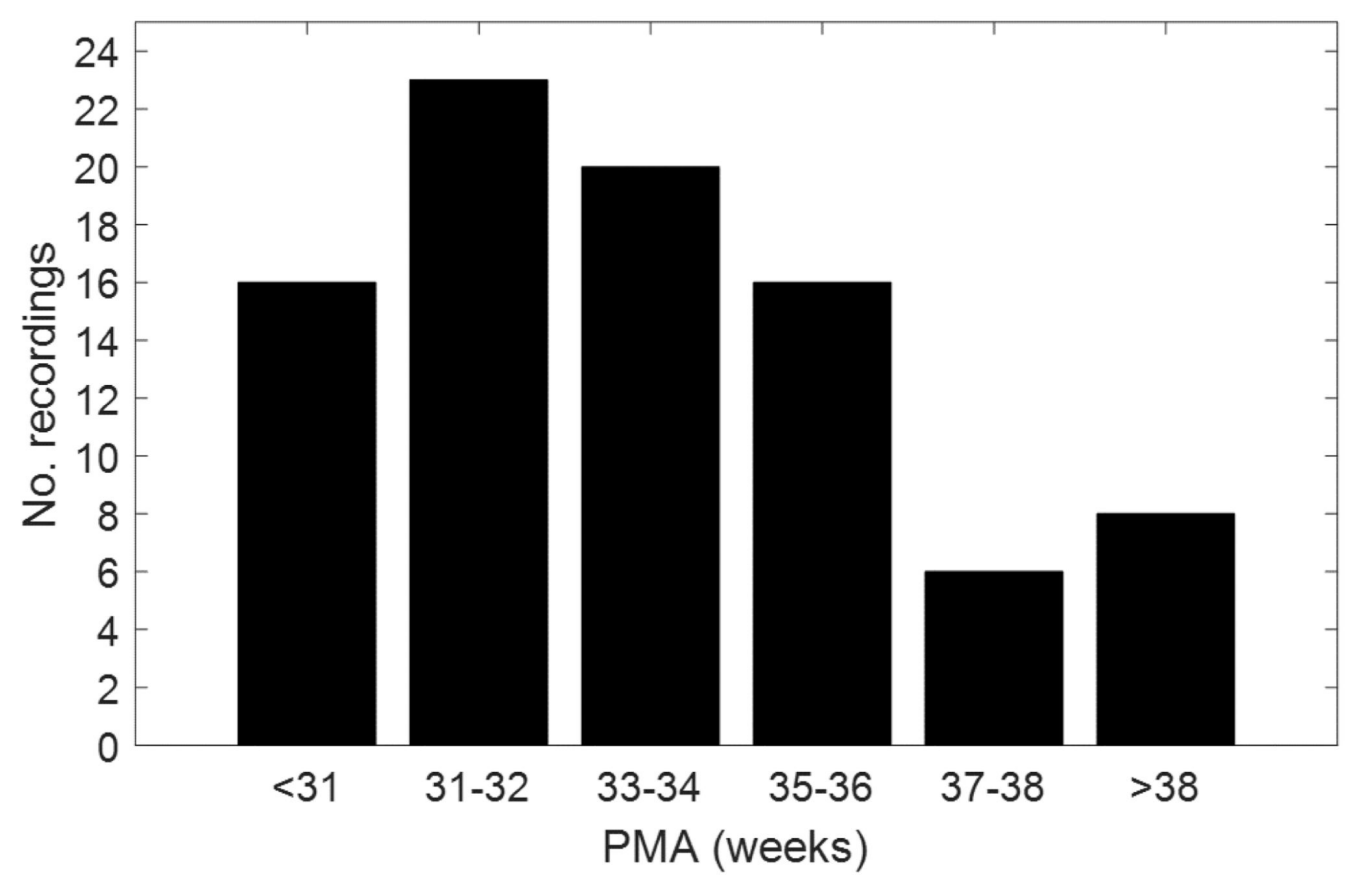
Histogram of the total number of EEG recordings used in the study, ordered by PMA. There are a total of 89 recordings ranging from 27 to 42 weeks PMA.

EEG recordings were obtained from the neonates between the first and the third week of life, followed by one recording every 2 to 3 weeks up to transfer or discharge. This resulted in 89 recordings ranging from 27 to 42 weeks PMA. The age distribution of this dataset is presented in the histogram of Fig. 1.

Mean EEG monitoring time was 4 h 55 min (range 1 h 40 min–9 h 00 min), in accordance with neonatal EEG surveillance guidelines^28^ to acquire at least two complete sleep cycles. Feeding and care were carried out per the normal routine of the NICU. Kangaroo Care was encouraged and allowed during the recordings as part of the application of the Newborn Individualized Developmental Care and Assessment Program. All EEG recordings were recorded with nine electrodes (Fp1, Fp2, C3, C4, T3, T4, O1, O2, and reference electrode Cz) placed per the modified international 10–20 standard locations (BRAIN RT, OSG equipment, Mechelen, Belgium) at a sampling frequency of 250 Hz. In premature infants <36 weeks PMA, unobtrusive sleep EEG monitoring was performed including a channel for respiratory activity, electrocardiogram and oxygen saturation. Infants ≥36 weeks PMA had an overnight PSG recording with 12-channel EEG, electrocardiogram, oxygen saturation, electromyogram, 2 electro-oculograms, piezoelectric belts (to measure abdominal and thoracic respiratory effort), and a nasal thermistor (for airflow monitoring before discharge).

In the remainder of this paper, the first 34 visually labeled recordings that were obtained for algorithm development and optimization, are referred to as the training set. The subsequent 55 labeled recordings obtained were referred to as the test set, used solely to assess final algorithm performance.

### 2.2 EEG visual sleep labelling

Video-EEG segments were visually related to different sleep states for the given PMA, by two independent EEG readers (AD and KJ), for periods of AS, QS, indeterminate sleep, and wakefulness. Sleep was defined based on previous definitions of EEG characteristics in premature sleep and simultaneous assessments of multiple cerebral and noncerebral measures were used to better identify neonatal state transitions. Physiological parameters of REM (present in AS, absent in QS), body movements (present in AS, absent in QS) and cardiorespiratory regularity (regular during QS, irregular in AS) were considered, depending on the behavioral state for the given PMA. Indeterminate sleep was defined as a sleep state with noncerebral characteristics of AS, coinciding with EEG features of QS, or vice versa, often observed in a transition from one state to another.^12^,^28^–^31^ In this study, the onset of QS or AS was considered as the beginning of a segment in which three consecutive minutes or three of four consecutive minutes were scored as QS or AS, respectively.^32^,^33^ Disagreed epochs and epochs with more than 3 min difference in overlap were re-evaluated and a final state was assigned based on consensus agreement. For the current analysis, AS, indeterminate sleep and wakefulness were grouped together as a single non-QS state, and the EEG finally categorized as either QS or non-QS. Cohen’s Kappa for inter-rater agreement of QS versus non-QS periods was calculated and proved to be high with Kappa = 0.93 (95% CI: 0.90–0.95)^34^ across all ages. The lowest inter-rater agreement was observed at the youngest ages <31 weeks PMA, with Kappa = 0.89 (95% CI: 0.82–0.96), and improved towards term ages.

### 2.3 EEG pre-processing

Data was band-pass filtered at 1–40 Hz, with an additional 50 Hz notch filter to remove mains noise. Electrode drop-off (the poor contact of an electrode) was also present in some recordings, in which case affected channels were discarded when >20% of the signal was missing.

### 2.4 Cluster-based adaptive sleep staging

**Fig. 2:**
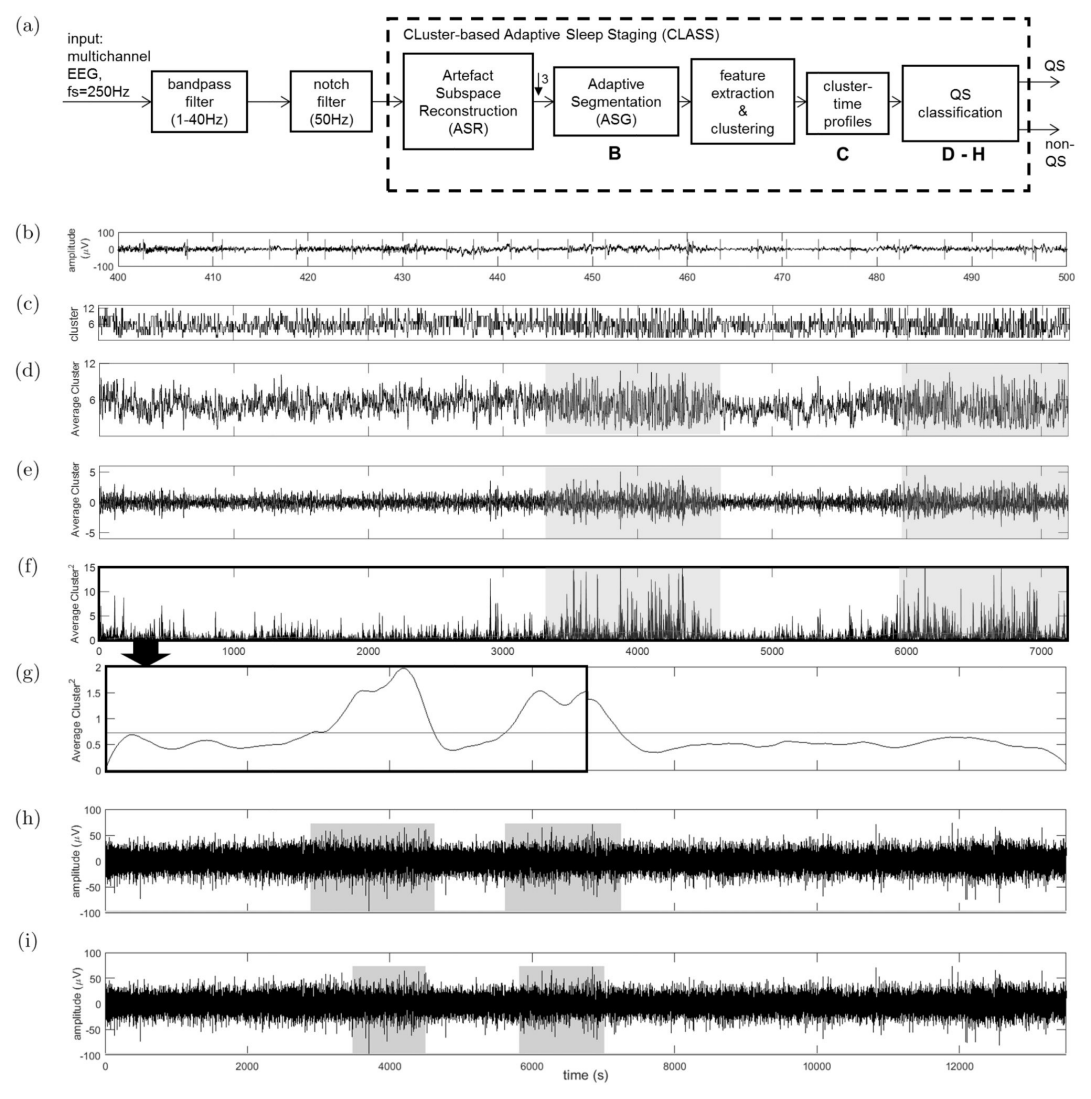
(Color online) (a) Flowchart of the stages of EEG processing by CLASS. (b) Illustration of the Adaptive Segmentation (ASG) stage for a 100 s period of EEG in a single channel. Red line denote the ASG segment boundaries. (c) Illustration of a Cluster-Time Profile for a 2 h epoch of EEG from a single channel. Features are extracted from each segment defined by ASG and then clustered and the corresponding segment cluster labels are then plotted over time for each sample. (d) The average cluster-time profile determined by taking the mean profile across all channels. Regions of increasing cluster fluctuation (shaded) correspond to higher EEG discontinuity and QS periods. (e) De-trended signal after subtraction of the average channel from its running mean. (f) The square of the zeroed signal with the signal envelope shown by a red curve. (g) The signal envelope of a complete 4 h EEG recording, with the mean threshold to estimate the QS periods shown in red. Here, the 4 h signal envelope is formed by stitching the signal envelope processed for every 2 h epoch of EEG. The first 2 h of the stitched envelope shown in this figure correspond to the envelope derived in (f). (h) The QS periods as estimated by CLASS after thresholding with the mean of the signal envelope. Estimated QS periods are shaded. (i) The shaded QS periods as visually estimated by the clinician using the full PSG recording.

CLASS assumes that QS is relatively more discontinuous than non-QS and that this is maintained over a wide range of PMA. The method extends concepts introduced by Krajča *et al.*^25^–^27^ and a flowchart of the algorithm stages are presented in Fig. 2(a). Each stage of the algorithm is detailed below in Secs. 2.4.1–2.4.4, with a series of parameters defined (in *italics*) throughout. The optimization method and the selected values for these parameters are presented in Sec. 2.5.

#### 2.4.1 Artifact subspace reconstruction

As CLASS aims to detect EEG discontinuities, it can easily confuse high power artifacts as periods of discontinuity and QS, and thus a rigorous artifact removal scheme was required.

**Fig. 3:**
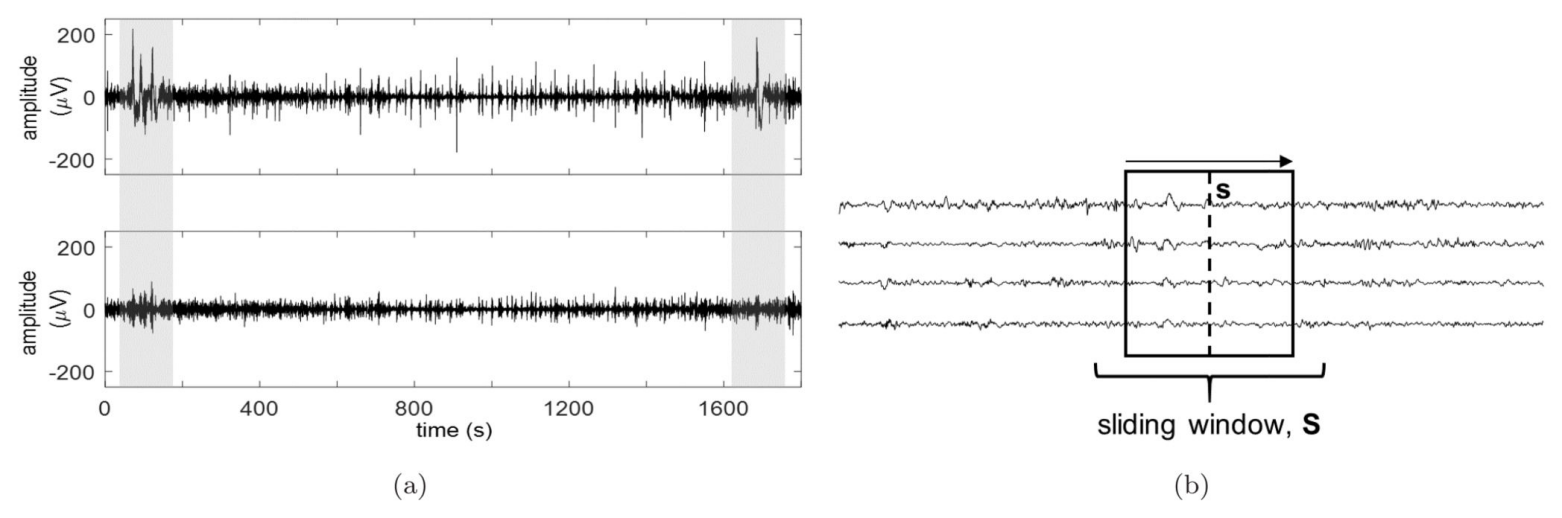
Illustration of the ASR method. (a) Top: A 30-min epoch of bandpass filtered (1–40 Hz) EEG in a single channel, before ASR is applied. High power artifacts are shaded. Bottom: The bandpass filtered signal after ASR is applied. The same shaded artifacts are now reduced while surrounding clean periods of the signal remain intact. (b) Illustration of the cleaning procedure of ASR on the EEG recording. Reconstruction metrics are calculated within the sliding window **S** in order to clean the sample of data along the dotted line denoted by **s**. As the sliding window moves sample-by-sample across the recording, the metrics are updated and the new sample **s** is cleaned.

Current neonatal EEG pre-processing often exclusively uses band pass and notch filtering for artifact removal.^24^ However, some artifacts cannot be sufficiently removed by this method alone. Popular artifact removal methods include Independent Component Analysis^35^ and Principal Component Analysis (PCA),^36^ which involve transforming the EEG channels into a new component space that more clearly isolates the artifacts. However, such methods assume that movement artifacts are also stationary in nature, which is not the case. An alternative technique called Artifact Subspace Reconstruction (ASR), developed by Kothe and Makeig,^37^ was used here. The method applies PCA over a sliding window along the EEG channels, locally separating high-power artifacts from the clean signal.^38^ ASR is illustrated in Fig. 3(a) on an epoch of EEG containing high-power, nonstationary artifacts.

ASR begins with a calibration procedure, where a 1 min epoch of artifact-free multichannel EEG (calibration data) is used to obtain thresholds for identifying clean signal and artifact subspaces within each sliding window of the EEG recording. With the subspaces identified, only the clean subspace is then used to reconstruct the signal.

A choice of calibration data at 40 weeks PMA was found, by trial and error, to best remove artifacts across the training set. To obtain the thresholds, PCA is performed on the calibration data in a robust manner by estimating the covariance matrix (**Y**) using the geometric median. With **x_i_** denoting the vector of calibration data amplitudes across channels at the *i*th time point, and *n* denoting the length of the calibration data, the geometric median (covariance) is defined by:

(1) $$\underset{\text{Y}}{\text{argmin}}\sum_{i=1}^{n}{\left\|{\text{x}}_{\text{i}}{\text{x}}_{\text{i}}^{⊤}-\text{Y}\right\|}_{2}.$$

Unlike the conventional (mean) covariance, **Y** is less skewed by the presence of possible residual artifacts that may not have been identified when the calibration data was first selected. After performing PCA on the calibration data (using the eigenvectors of **Y**, defined as **V_Y_**), each resulting principal component is segmented into fixed-length segments and the root-mean-square (RMS) power calculated for each segment. 0.5 s is chosen as the segment duration, to match the typical time length of discontinuities in the signal, and produce enough windows to calculate a smooth RMS distribution. 66% window overlap is used to avoid missing any discontinuities at the segment boundaries. A Gaussian distribution is fitted to the RMS values of each component, and the component threshold (*t_c_*) is defined based on the mean (*µ_c_*) and standard deviation (*σ_c_*) of the fitted distribution:

(2) $$t_{c}=\mu_{c}+\text{ASR\_thresh}\cdot \sigma_{c}.$$

ASR_thresh is a parameter for weighting the contribution of the standard deviation. The choice of estimating *µ_c_* and *σ_c_* from a Gaussian, rather than directly from the RMS values, is to further ensure the robustness of the threshold estimates to potential extremities in the RMS distribution (brought upon by residual artifacts). The resulting set of component thresholds (**t** = [*t*_1_ *t*_2_ … *t_c_* …]) is represented as a diagonal threshold matrix (**T**). In addition to **T**, a mixing matrix (**M**) is also defined in this calibration stage, from the covariance matrix (by **Y** = **MM^⊤^**). **M** is required to later reconstruct the EEG signal from the identified clean subspace.

To identify the clean and artifact subspaces from an EEG window (**S**) in the recording, conventional PCA is applied to **S**, by obtaining the eigenvector (**V**) and eigenvalue (**Λ**) matrices from the window’s covariance matrix (by **Σ** = **VΛV^⊤^**). Determining which of these EEG window’s principal components are potential artifacts is achieved by comparing **Λ** to **T**, after **T** is first projected into the same principal component space as **Λ**. Projecting **T** is achieved by returning it from the calibration principal component space, which it was first defined in, to the original EEG space (using the calibration eigenvectors **V_Y_**). This is then re-projected from the EEG space to the new principal component space of **Λ** (using the window’s eigenvectors **V**):

(3) $${\text{T}}^{\text{proj}}={\text{TV}}_{\text{Y}}^{⊤}\text{V}.$$

The resulting projected thresholds (**T^proj^**) is a full matrix representing the RMS thresholds for each of the window’s principal components, while the diagonal matrix **Λ** is equivalent to the total variance along each principal component. As the original EEG window is zero-mean (achieved by the band-pass filter during pre-processing in Sec. 2.3), the variance is equivalent to the square of the RMS. Therefore, the eigenvalues can be directly compared to the thresholds, by squaring each element of **T^proj^** and summing the resulting variances along each column (each principal component) to achieve the total threshold variance for each component, as shown in (4) below. This forms a binary matrix (**A**) which identifies those components that lie below the threshold (the clean subspace) and those that form the artifact subspace, by setting each *j*th row of **A** (denoted by a_j*_ below) to ones or zeros, respectively:

(4) $${\text{a}}_{\text{j*}}=\{\begin{array}{ll} 1, & \lambda_{jj}<\sum_{i}t_{ij}^{2},\\ 0, & \lambda_{jj}\geq \sum_{i}t_{ij}^{2}, \end{array}\Lambda =\left\{\lambda_{ij}\right\},{\text{T}}^{\text{proj}}=\left\{t_{ij}\right\}.$$

The final step to reconstruct the EEG from the clean subspace, is performed at a fixed time point (sample) within the EEG window, with this vector of amplitudes across channels denoted by **s**. This is illustrated in Fig. 3(b). The previously determined mixing matrix (**M**) and **s** are rotated into the same space as **A**, spanned by the window’s eigenvectors (resulting in **V^⊤^M** and **V^⊤^s**, respectively). This rotation allows a ‘reduction’ of **V^⊤^M** directly by **A** (as **V^⊤^M** ◦ **A**, where ◦ denotes elementwise multiplication), with the result used to perform a clean, linear projection of the rotated sample **V^⊤^s**. This projection is clean, as **V^⊤^M** ◦ **A** removes the contribution of the artifact subspace. By finally reversing this clean projection (using the full **V^⊤^M**) and rotating back to the original EEG space, the cleaned EEG sample (**s_clean_**) is reconstructed from only the clean subspace. These operations simplify into a single reconstruction matrix (**R**) for cleaning **s**, with + denoting the pseudo-inverse:

(5) $$\text{R}=\text{M}{\left({\text{V}}^{⊤}\text{M}\circ \text{A}\right)}^{+}{\text{V}}^{⊤},$$

(6) $${\text{S}}_{\text{clean}=\text{Rs}.}$$

To clean the entire EEG, the sliding window is shifted sample-by-sample along the recording, with **R** recalculated each time and applied to the new **s** to obtain **s_clean_**. **T** and **M** (used for determining **R**) are both derived from the calibration sequence and remain the same throughout.

#### 2.4.2 Adaptive Segmentation

To reduce processing time after ASR is applied, the cleaned recordings are downsampled by a factor of three, reducing the sampling frequency to 83 Hz (while satisfying the Nyquist rate of the band-pass filtered (1–40 Hz) EEG).

Each EEG channel is divided into varying length segments (typically 1–5 s long) by Adaptive Segmentation (ASG), which segments the signal nonuniformly such that each segment locally resembles a specific EEG pattern and characteristic.

ASG utilizes sliding contiguous windows comparing amplitude and frequency-based measures between the windows as they slide along the recording, to detect periods where large changes occur.^26^ The locations of these large changes denote ASG segment boundaries. This reflects where deviations in both the amplitude and frequency behavior exist, and the onset of a new segment pattern.

Each window has length *WIN* seconds which moves along the channel in steps of *SHIFT* samples. For each shift, the amplitude-based and frequency-based measure is calculated for each window. Denoting ADIF and FDIF as the amplitude- and frequency-based measures, respectively, and *x*(*i*) as the *i*th sample in the window:

(7) $$\text{ADIF}=\sum_{i=1}^{\text{WIN}}\left|x(i)\right|,$$

(8) $$\text{FDIF}=\sum_{i=1}^{\text{WIN}}\left|x(i)-x(i-1)\right|.$$

Differences between the measures from each window are determined and combined in a weighted difference measure (*G*), with the subscripts 1 and 2 denoting which window the measures originate from:

(9) $$G=\left|{\text{ADIF}}_{1}-{\text{ADIF}}_{2}\right|+kF\left|{\text{FDIF}}_{1}-{\text{FDIF}}_{2}\right|.$$

*kF* is an integer parameter that weighs the contribution of the FDIF (and ADIF) measures.

Calculating *G* for every shift along the channel results in the signal *G*(*t*) over time for the full EEG recording. The ASG segment boundaries are estimated from the peaks of *G*(*t*). From *G*(*t*), it was noticed that oversegmentation could occur due to too many low-amplitude peaks, making the algorithm computationally expensive. Additions to this method have previously been attempted to solve this issue, such as incorporating a static or adaptive peak threshold.^26^ Here, we choose to modify the method using a pair of thresholds that set a minimum allowable height (*MINPEAKHEIGHT*) and distance (*MINPEAKDISTANCE*) between successive peaks.

ASG is applied separately for each EEG channel and Fig. 2(b) shows the segment boundaries for a 100 s epoch of EEG in a single channel.

#### 2.4.3 Feature extraction and cluster-time profiles

After segmenting the EEG into distinct characteristic segments by ASG, clustering is performed to group similar segments together. Using variance alone to group these segments is sensitive to artifacts and other unusual amplitude fluctuations, while not fully expressing the distinct behaviors between them. Thus, the defined characteristics of each group can be more distinctly expressed by calculating a series of both time-domain and frequency-domain EEG features:

- Amplitude Standard Deviation
- Difference between maximum and minimum amplitudes
- Maximum absolute amplitudes of first derivative of samples
- Maximum absolute amplitudes of second derivative of samples
- Mean frequency of EEG activity
- Square root of the power in the delta (1–3 Hz) frequency band
- Square root of the power in the theta (3–8 Hz) frequency band
- Square root of the power in the alpha (8–12 Hz) frequency band
- Square root of the power in the beta (12–30 Hz) frequency band

The mean frequency and power measures are calculated from the periodogram of power spectral density.

The features (and therefore the corresponding segments) from all channels are clustered together into *k* clusters using the *k*-means algorithm (with 20 repetitions to ensure a good initialization and clustering performance) and the mean variance of each clustered group of segments used to relabel the clusters by increasing order. Each sample in a segment is then replaced by its cluster label and plotted over time^27^,^39^ and the resulting label evolution over time for all channels are referred to as cluster-time profiles. A cluster-time profile of a 2 h EEG recording is shown in Fig. 2(c) for a single channel.

#### 2.4.4 QS classification

By representing the evolving characteristics of the segments using cluster-time profiles, periods indicating large changes in segment behavior (the relatively higher discontinuity associated with QS) are reflected by larger fluctuations in the cluster labels. To classify these periods as QS:

(1) The cluster-time profiles are averaged across channels forming a single average cluster-time profile. This is to accentuate periods of large fluctuation, while smoothing out channel-specific deviations and is illustrated in Fig. 2(d). Periods of relatively larger fluctuation (evident during QS) are also shaded.
(2) The resulting average profile is de-trended by subtracting the running (time-varying) mean of the profile (Fig. 2(e)). This eliminates any natural underlying transients in the signal that may affect the QS classification. The running mean is calculated using a moving average (MA) filter of length *avg_win_length* samples and the resulting profile squared to further accentuate the peak regions (Fig. 2(f)).
(3) A longer MA filter of length *smooth_win_length* samples is further applied to the squared profile to produce a smooth envelope curve. QS periods are estimated by a threshold, calculated as the mean of the envelope curve (Fig. 2(g)).

In cases of long recordings >2 h are processed (common in preterm recordings >36 weeks PMA), envelopes are derived separately for each 2 h segment and then stitched together before thresholding is performed (as in Fig. 2(g)).

A minimum of three consecutive minutes or three out of four consecutive minutes of the same sleep state are required to identify AS and QS, as described in previous studies.^33^,^40^ Based on this scoring criteria, QS detections <3 mins are removed as a final post-processing stage. Figure 2(h) shows the output of CLASS with the estimated QS periods shaded, and the corresponding clinicians’ visual QS labels are shown in Fig. 2(i).

### 2.5 CLASS Optimization

The CLASS parameters (*ASR_thresh, WIN*, *SHIFT, kF, MINPEAKHEIGHT, MINPEAK DISTANCE, k, avg_win_length*, and *smooth_win_length* presented throughout Sec. 2.4) required specific tuning to perform best across the full PMA range. A summary of the parameters and definitions can be found in Table 1.

For parameter optimization, an exhaustive grid search is often used, where all possible combinations of parameters are tried with the algorithm to achieve a global optimum. However, with many parameters to tune, such a procedure would be computationally expensive. In addition, performance needed to be assessed over a range of PMAs. Selecting a single optimization criterion over age (as is typical for a grid search) was not appropriate for assessing age specific changes as the parameters were varied. Furthermore, certain combinations of parameters could cause the algorithm to become detrimentally slow and inefficient. Therefore, perturbation analysis was used to determine a sufficiently good set of parameters, using the defined training set of recordings, aged 27–40 weeks PMA.

In perturbation analysis, parameters are initially selected based on methods in literature^25^–^27^ and informed estimates. Each parameter is independently perturbed in large steps and updated if it clearly improves CLASS performance (along with reasonable computational efficiency), based on Sensitivity and Specificity, when compared to the clinicians’ visual labeling. Those parameters whose CLASS performance is sensitive to, are additionally tuned using a finer local sweep to further improve performance. The optimized CLASS parameter values, as selected by perturbation analysis, are also listed in Table 1. Parameters which proved most sensitive to CLASS performance are indicated.

### 2.6 Measures of agreement and assessing CLASS performance

Based on those ages exhibiting similar EEG behavior, six groups were defined according to PMA^28^,^41^ spanning two weeks (group 1: <31 weeks, group 2: 31–32 weeks, group 3: 33–34 weeks, group 4: 35–36 weeks, group 5: 37–38 weeks, group 6: >38 weeks). Agreement of the clinicians’ visual labeling and CLASS-estimated QS periods was initially determined by the Sensitivity and Specificity. While these measures assess agreement between visual labeling and CLASS labeling sample by sample, they do not specify exactly how many QS periods are correctly detected or the exact number of misclassifications. We use two additional measures to quantify this, the Detection Factor (DF) and Misclassification Factor (MF). DF measures the proportion of visually labeled QS periods correctly detected by CLASS (also referred to in literature as the True Positive Fraction^42^), while MF measures the proportion of the CLASS-detected periods that are misclassifications:

(10) $$\text{DF}=\frac{\text{No.}\;\text{of}\;\text{correctly}\;\text{detected}\;\text{periods}}{\text{Total}\;\text{no.}\;\text{of}\;\text{visually}\;\text{labeled}\;\text{periods}},$$

(11) $$\text{MF}=\frac{\text{No. of incorrectly detected periods}}{\text{Total}\;\text{no.}\;\text{of}\;\text{CLASS}\;\text{detected}\;\text{periods}}.$$

Both measures, being a proportion, have a range 0–1. A correctly detected QS period was defined if the CLASS-estimated and visually labeled period overlapped by >50%.

As an overall measure of performance, Receiver Operating Characteristics (ROC) curves and AUC values were defined across the test recordings (using the optimized parameters). The classification threshold of the smooth envelope curve (the red line shown in Fig. 2(g)) was varied about the originally selected mean value, for each recording. The median ROC curve over the recordings was then determined and its area under curve (AUC) calculated by the trapezium integration method. Median values were selected, as with most measures defined in this study, in the case of any extreme values brought about by analyzing over a wide range of PMA. CLASS performance was further assessed with respect to PMA over the range 27–42 weeks, using Sensitivity, Specificity, DF and MF.

These agreement measures were also calculated for CLASS without ASR (using band-pass filtering alone), to assess the importance of ASR. Similarly, to quantify the contribution of ASG, CLASS was run alternatively using a uniform segmentation of the EEG signal at both 1 s and 5 s durations (the typical duration range of the adaptive segments). CLASS was also applied using only the segment standard deviations to derive the profiles and classify the QS periods, omitting the calculation of multiple features and the clustering stage.

As a third and final assessment of performance, CLASS was compared to the SAT% algorithm of Palmu *et al.*^24^ The algorithm is based on the Non-Linear Energy Operator (NLEO). With *x*(*i*) denoting the *i*th sample of the EEG channel, NLEO is defined as:

(12) NLEO(i)=x(i)×(i−3)−x(i−1)×(i−2).

To classify the QS periods in each recording, a threshold was applied to the final SAT% signal, defined as the mean SAT% of the recording. This allowed the threshold to change for each recording, adapting to the potential change in SAT% behavior with PMA. The threshold was selected by assessing classification performance on the training set (compared to visual labeling) under different weighted mean values, with the conventional mean performing best.

### 2.7 Defining normal maturational trends

To illustrate the usefulness of automated QS detection by CLASS in assessing electro-cortical brain development, QS characteristics were derived from the CLASS QS estimates on the test set to obtain QS-specific maturational trends. This was performed on the band-pass filtered EEG to allow for a direct comparison of the trends with those from the clinicians’ visual QS labeling.

Scher *et al.* and Jennekens *et al.* have previously revealed maturational trends in the spectral powers of preterm cohorts,^40^,^43^ while Koolen *et al.* have developed a robust burst detection method to assess the change in burst behavior in EEG over age.^18^,^44^ Based on these previous findings, the following characteristics were calculated for defining the trends:

(1) Relative spectral power in the delta, theta, alpha, and beta energy bands, calculated by dividing each band power by the total power over the full frequency range. Relative values take into account between-subject variability in total spectral power, which may vary substantially due to slightly different electrode positions between recordings.
(2) Burst percentage (Burst%), to quantify the relative proportion of suppressed periods (interburst intervals (IBIs)) and bursts in the signal. This used a robust burst detection method developed by Koolen *et al.*^44^

Median characteristics over channels were calculated from the QS periods to improve robustness to channel-specific deviations. The mean characteristic value across all QS periods in an individual recording was then used to test for a significant correlation with PMA. To correct for intra-patient and inter-patient variability, a random effects regression model was selected and extended to test for nonlinear trends. Statistical analysis was performed in SPSS version 23.

As well as comparing with the visual QS trends, CLASS QS trends were also compared to those derived from nonstate specific periods of EEG (both QS and non-QS). This was to determine if QS-specific trends were more clearly defined, and there-fore warranted. Nonstate specific EEG periods were extracted by selecting 20 min successive epochs of EEG (equivalent to the average QS duration) across up to 4 h of EEG, depending on the total recording length. These extracted epochs were used to derive similar trends as for the QS periods.

## 3 Results

### 3.1 Assessing CLASS performance on the test set

#### 3.1.1 CLASS performance with respect to PMA

**Fig. 4:**
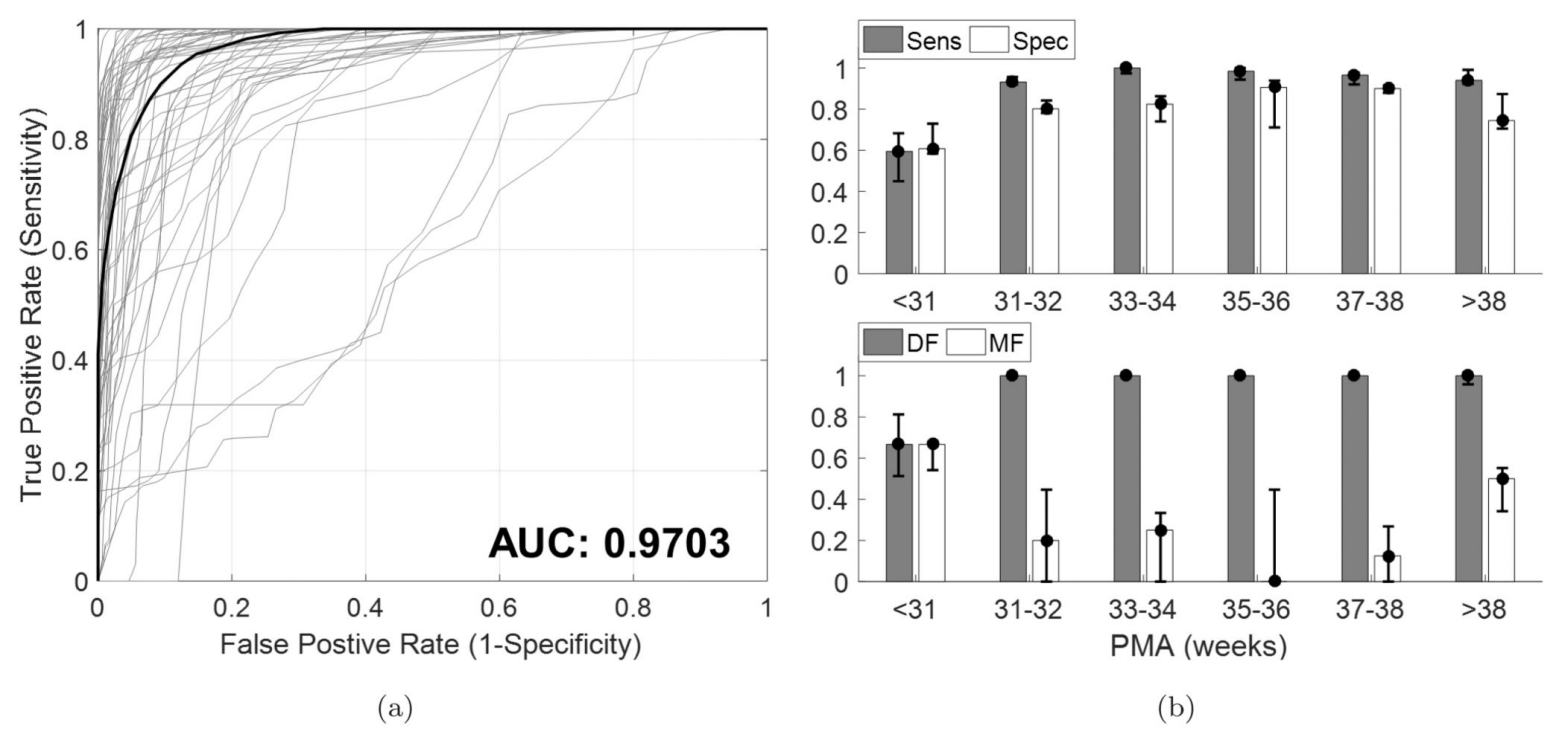
Assessing the performance of CLASS on a test set of 55 recordings aged 27–42 weeks PMA. (a) ROC of CLASS performance by varying the detection threshold while keeping all other optimized parameters constant. ROC curves for each recording in the test set (in gray) are shown, and resulting median ROC curve (in black). The AUC of the median ROC curve is also presented. (b) CLASS performance with respect to PMA denoting Sensitivity (Sens), Specificity (Spec), DF and MF. DF and MF denote Detection Factor and Misclassification Factor measures, respectively. DF measures the proportion of visually labeled QS periods correctly detected by CLASS, while MF measures the proportion of CLASS-detected periods that do not correspond to the visual QS periods (i.e. are misclassifications). Error bars denote the medians and IQRs.

Figure 4(a) shows the overall ROC performance of CLASS on the test set for each recording (in gray) and the median ROC curve (in black) which has an excellent AUC of 0.9703.

Figure 4(b) shows the Sensitivity, Specificity, DF and MF results for CLASS over PMA. Error bars denote the medians and interquartile ranges (IQRs).

In preterm infants in the range 31–38 weeks PMA, CLASS distinguished QS periods with excellent Sensitivity (median Sensitivity range 0.93–1.0), DF (median DF = 1), Specificity (median Specificity range 0.80–0.91), and MF (median MF range 0–0.25). Between 35–36 weeks PMA, MF was optimal (median MF = 0) indicating very few misclassifications. At >38 weeks PMA, while DF, Sensitivity, and Specificity remained high, MF was also comparatively higher than at younger ages (median MF = 0.50). This suggested that QS periods were well detected but misclassifications were also prevalent. For ages <31 weeks, CLASS performance was most dubious, showing comparatively worse results for all measures. Recordings <31 weeks PMA corresponded to the poorer ROC curves shown in Fig. 4(a), although these were few. Overall, the results show that CLASS has an affinity to EEG recordings in the range of 31–38 weeks PMA.

#### 3.1.2 Comparing CLASS performance for different algorithm stages

Table 2 lists the median Sensitivity, Specificity, DF, MF and AUC values across recordings, comparing CLASS in its entirety to CLASS without ASR (CLASS-noASR), CLASS with uniform segmentation of 1 s (CLASS-USG1), CLASS with uniform segmentation of 5 s CLASS-USG5), CLASS using only standard deviation instead of multiple features and clustering (CLASS-SD), and the SAT% method. A paired *t*-test was used to test for statistically significant differences between CLASS and the other algorithms/versions of CLASS. Asterisks denote significant differences at the *p* < 0.05 level.

When comparing CLASS to CLASS-noASR, all agreement measures showed significant differences. Notably, CLASS-noASR had a higher MF of 0.40 and lower AUC of 0.85, although all measures were comparatively worse than CLASS with ASR. This shows the great importance of ASR in improving the quality and robustness of CLASS.

Comparing CLASS performance against CLASS-USG1, DF proved to be equivalent, with a statistically significant improvement in Sensitivity with CLASS-USG1 (although small). However, Specificity of CLASS-USG1 was significantly lower (0.76) compared to CLASS (0.82). Although not statistically significant, MF and AUC also showed lower values with CLASS-USG1 (0.33 and 0.96) compared to CLASS (0.25 and 0.98). Increasing the length of the uniform segmentation, as in CLASS-USG5, produced a worse performance. Apart from DF (which remained the same as CLASS), all other values were statistically significantly poorer than CLASS, including MF and AUC values (0.33 and 0.95).

Performance with CLASS-SD revealed no statistically significant differences. In fact, CLASS-SD resulted in a slightly better MF and Sensitivity value of 0.20 and 0.84 respectively, compared to CLASS (0.25 and 0.82). However, Sensitivity and AUC were both (marginally) lower than with CLASS.

#### 3.1.3 Comparing CLASS with SAT%

The final comparison is between CLASS and the SAT% algorithm. It is clear from the results of all measures that the SAT% method was poor at performing automated QS detection. This indicates that SAT% alone is insufficient for accurately and robustly detecting QS.

### 3.2 Assessing QS characteristics

Table 3 lists the regression analyses for the QS characteristics of Burst% and the relative spectral powers for delta, theta, alpha, and beta bands during QS. This is shown for CLASS, visually labeled QS and nonstate specific EEG periods. Regression analysis results are presented with a *p*-value (significance defined as *p* < 0.05), a coefficient *b* (slope of the regression line), standard error, and 95% confidence intervals.

Based on the optimal ages for CLASS performance identified from the results in Sec. 3.1, QS characteristics were scrutinized only for the PMA range of 31–38 weeks (resulting in 45 of the original 55 test recordings studied), as results for >38 weeks and <31 weeks would have proven unreliable due to the higher number of misclassifications.

After log-transformation, Burst% during QS (both with CLASS and visually labeled estimates) increased significantly with PMA (*p*< 0.001, linear correlation), and spectral power analyses showed a significant trend for relative delta, theta and beta powers during QS. Relative delta power decreased slightly across PMA, (*p*< 0.01 linear correlation), while relative theta power showed a significant quadratic relationship with PMA (*p* < 0.05). Relative alpha band power showed no significant correlation with PMA in both visual and CLASS QS estimates, whereas relative beta power showed a clear quadratic relationship (*p* < 0.05 CLASS, *p* = 0.056 visual). In terms of the slopes of the significant trends, all characteristics showed very similar agreement between CLASS and visual QS estimates, particularly for Burst% (*b* = 0.045 for both CLASS and visual) and log relative delta power (*b* = −0.014 for CLASS, *b* = −0.013 for visual). When compared with the maturational features derived from the non-state specific EEG epochs, the trends derived from QS proved to be superior. While the decrease in relative delta power and increase in Burst% with PMA were still significantly correlated, they were weaker, and relative theta and beta powers showed no significant correlations.

## 4 Discussion

To the best of our knowledge, this study provides the first approach for automated QS detection in multichannel EEG recordings of preterms without preselecting a PMA range. We show that the physiologically inspired CLASS algorithm can successfully and robustly capture detections of QS periods. The study also provides a preliminary illustration for objectively examining premature sleep behavior, based on the fully automated detection and quantification of normal maturational trends from QS periods, and the broad age range that can be targeted.

Automated detection of sleep is challenging during this period of rapid brain maturation, because of the biological and technical variability in EEG background patterns. Previous attempts were either based on limited channel EEG recordings in very preterm infants <32 weeks^22^,^45^ or focused on neonatal, term EEG.^23^,^40^,^46^–^48^ From a physiological point of view, the performance of CLASS actually reflects the development of QS EEG behavior in preterm infants, since it is based on the relative discontinuity at each PMA caused mainly by the difference in amplitude of the EEG activity between QS and other states.^28^,^41^

The overall results of the ROC, Sensitivity, Specificity, DF, and MF (with CLASS in its entirety) confirm the ability of this novel, automated algorithm to align with clinicians’ visual PSG sleep labeling and identifies the best performance of the algorithm to classify QS at 31–38 weeks PMA.

At <31 weeks, there is still great uncertainty in classifying QS. A combination of behavioral and EEG characteristics have been used for visual sleep labeling, since neither of these characteristics alone are considered as the gold standard.^12^,^28^,^33^,^49^,^50^ However, when all these criteria are required for state definition, increasingly immature infants will have higher proportions of indeterminate sleep. This indicates the immaturity of each cerebral and noncerebral sleep characteristic to represent distinct sleep states in the very premature infant^50^ resulting in an increase of indeterminate sleep periods and lower levels of definite QS.^11^,^20^,^50^,^51^ However, this limitation is true for visual as well as for CLASS classification. CLASS relies on discontinuity, and in this respect, indeterminate sleep can strongly resemble QS and be detected as such by the algorithm. Therefore, in premature infants <31 weeks, CLASS more accurately captures vigilance state cyclicity (variations in the states of discontinuity that is made up of both QS and indeterminate sleep periods) rather than definite QS, and should be interpreted as such.^11^,^20^,^22^,^45^,^51^

Near term age of >38 weeks PMA, a new sleep developmental trajectory is expressed, with the emergence of both high voltage slow-wave as well as *tracé alternant* QS patterns. This leads to a globally more continuous EEG and the relative change in discontinuity between QS and non-QS becomes less distinguishable. At this point, misclassifications may be too intrusive within the signal, further explaining the higher MF values and lower Specificity for infants >38 weeks PMA.

Introduction of the artifact removal method, ASR, proves to remove a major proportion of artifacts, as revealed in the improvement in results (most clearly notable with MF) when included. Of all the stages of CLASS (ASR, ASG, clustering), results point to ASR as providing the most significant improvement in performance. High power artifacts can skew the clustering by CLASS and ‘appear’ to be discontinuous QS, while also affecting the signal stitching. Upon division into 2 h segments, regions containing artifacts can be classified differently to regions that are relatively artifact-free. Envelope peak amplitudes may differ across stitched segments as a result, and the QS detection threshold becomes highly inaccurate. With this said, the use of a single age calibration sequence might still be a limitation of ASR. In future applications of CLASS, the ability of ASR to automatically and robustly select a sequence over PMA, and therefore adapt to age, may further improve the QS detection capabilities of CLASS at the extreme ages.

The use of ASG as opposed to a uniform segmentation also results in better detection. The separation of the EEG into characteristic segments allows for a more structured and distinct clustering, unlike the selection of segments achieved by uniform segmentation. Such an arbitrary selection at fixed time points can lead to adjacent segments with elevated cluster labeling, resulting in increased misclassifications (the rise in MF) when compared to ASG. In addition, CLASS-USG1 was more computationally expensive than CLASS as it resulted in a larger number of segments to process by the algorithm. However, we showed that simply increasing the length of the segmentation (to 5 s) to alleviate this, is further detrimental to performance. Overall, this indicates that ASG improves CLASS performance, providing a faster segmentation of the EEG that will aid in the algorithm’s clinical usefulness.

The use of SD only for QS classification, yields similar results to CLASS with multiple features and clustering. While SD is typically a very sensitive feature to high-power artifacts, with the inclusion of ASR and ASG in preceding steps, it proves sufficiently robust within this dataset to classify QS well. Therefore, this implementation of CLASS may be sufficient in most cases and is a more intuitive interpretation of the method. However, when dealing with very noisy recordings (that may not be adequately filtered by ASR), SD would be more susceptible to artifacts than using a combination of features (and clustering), for distinguishing between QS and non-QS. Encountering noisy recordings is especially likely when assessing clinical outcomes from very large EEG datasets with no preselection and limited screening.

Comparison of CLASS with SAT% further motivates the usefulness and novelty of CLASS in the clinical setting. SAT% depends on the detection of the suppressed periods of EEG (the IBIs) in the EEG signal (with periods of longer IBI duration resulting in lower SAT% values). However, as the preterm matures, IBIs are reduced and effectively vanish near term age. The method is therefore only feasible at very young ages, although even then, SAT% more accurately resembles vigilance state cyclicity^22^,^45^ rather than definite QS (as in the case of CLASS). Therefore, while SAT% continues to show merit in other research, its use in explicit QS detection culminates in a poor performance across all ages.

To demonstrate the usefulness of CLASS for studying maturational trends, we show that QS-derived characteristics are very similar between visual and automated assessment. When focusing on the age groups with the best performance of CLASS (31–38 weeks PMA), the findings of time-domain and frequency-domain characteristic trends in these selected QS periods agree with those previously reported, and prove to be stronger than those assessed on nonstate specific EEG periods.^17^,^31^,^43^,^52^ Furthermore, with the close agreement in maturational trends between CLASS and visual QS estimates, the algorithm allows for the complete automation of this entire process. Future work with the aid of CLASS may help to define the neurophysiological basis for background alterations in QS, and determine different maturational trends in infants with abnormal brain maturation.

Some limitations to this study are recognized. Our aim to develop a novel automated QS detection algorithm required the selection of a well-characterized dataset of healthy premature infants. As a consequence, this resulted in a small sample size with limited recordings, especially in the youngest and oldest PMA groups (further affected by the use of a distinct training and test set). However, to assess the robustness of the algorithm and avoid possible bias due to preselection of data, all EEG recordings were included (without omission), making this study transparent.

To assess algorithm performance, accurate visual sleep classification is also required. Cerebral and noncerebral signals were used in combination to increase the accuracy, but this ground truth remains somewhat ambiguous. Recently, the American Academy of Sleep Medicine^12^ renewed their recommendations for neonatal EEG sleep scoring in infants zero to two months of age. However, strict rules for scoring sleep from EEG in premature infants are lacking and based on expert opinion and most of the previously published studies of (automated) neonatal sleep classification, are from the experience of a single rater.^21^,^22^,^48^ As a first step to optimize visual classification (and the accuracy of the ground truth), two raters independently labeled the data for this study. In our opinion, the inter-rater agreement achieved in this study (Kappa = 0.93), was sufficiently high to use as a basis for algorithm development. However, testing on new well-described databases and the input of different EEG experts, will further improve preterm and term neonatal EEG sleep interpretation.

Developing a QS detection algorithm was chosen as a first step to fully automate the analysis of preterm sleep behavior, but we did not yet focus on AS detection. However, the importance of AS in the conservation of a qualitative sleep-wake cycle, cannot be overstated.^11^ Further directions towards algorithm development will aim to implement automated AS detection together with QS detection.

## 5 Conclusion

This is the first study to automatically and robustly detect QS periods from EEG recordings of preterm infants, covering a wide range of PMA (well into the final trimester of human pregnancy). The introduction of ASR to the CLASS algorithm improves robustness to artifacts in long duration multichannel EEG recordings, and most significantly strengthens the direct practical applicability of CLASS to aid clinical care. Objective QS maturational trends from CLASS QS estimates agree with the clinician’s visual labeling and provides stronger trends than those derived from nonstate specific EEG periods in the recordings. This opens the possibility for fully automated detection of abnormal preterm brain maturation and allows for further exploration into the relationship between cerebral activity, brain development, and neurodevelopmental outcome.

## Figures and Tables

**Table 1:** CLASS parameters that are tuned by perturbation analysis.

Parameter	CLASS stage	Definition	Tuned value
*ASR_thresh*	ASR	Threshold for separating the artifact and artifact-free subspaces in the EEG.	10
*WIN*^a^	ASG	Length of the contiguous windows that slide across the EEG. Used to detect large amplitude and frequency changes in the signal for identifying adaptive segment boundaries.	0.7s
*SHIFT*^a^	ASG	The step shift size of the sliding contiguous windows.	9 samples
*kF*	ASG	The weighting used to determine the joint contributions of the frequency and amplitude measures from which adaptive segment boundaries are determined.	10
*MINPEAKHEIGHT*	ASG	Minimum height between peaks in the combined amplitude and frequency signal, for defining an adaptive segment boundary.	100
*MINPEAKDISTANCE*^a^	ASG	Minimum allowable distance between successive adaptive segment boundaries.	25 samples
*k*	Feature Extraction and Clustering	Number of clusters for grouping the features used to define the cluster-time profiles.	12 clusters
*avg_win_length*	QS classification	Window length of moving average filter to determine a running mean of the cluster-time profile, for de-trending the signal.	500 samples
*smooth_win_length*^a^	QS classification	Window length of moving average filter to smoothen the cluster-time profile signal for QS classification.	35,000 samples

*Note*: CLASS: Cluster-based Adaptive Sleep Staging (automated QS detection algorithm); ASR: Artifact Subspace Reconstruction; ASG: Adaptive Segmentation; QS: Quiet Sleep.

a denotes CLASS-sensitive parameters which caused large changes to the performance of the algorithm, when fluctuated.

**Table 2:** Comparing CLASS performance at different stages of the algorithm, using Sensitivity (Sens), Specificity (Spec), DF and MF.

Algorithm	Median Sens (IQR)	Median Spec (IQR)	Median DF (IQR)	Median MF (IQR)	Median AUC (IQR)
CLASS	0.97 (0.92–1.0)	0.82 (0.71–0.88)	1.0 (1.0–1.0)	0.25 (0–0.5)	0.98 (0.92–0.99)

CLASS-noASR	0.81 (0.61–0.95)^a^	0.74 (0.65–0.83)^a^	1.0 (0.62–1.0)^a^	0.40 (0.25–0.65)^a^	0.85 (0.71–0.96)^a^
CLASS-USG1	1.0 (0.94–1.0)^a^	0.76 (0.67–0.82)^a^	1.0 (1.0–1.0)	0.33 (0–0.44)	0.96 (0.91–0.99)
CLASS-USG5	0.92 (0.87–0.98)^a^	0.79 (0.68–0.87)^a^	1.0 (1.0–1.0)	0.33 (0.036–0.54)^a^	0.95 (0.89–0.97)^a^
CLASS-SD	0.95 (0.90–1.0)	0.84 (0.73–0.90)	1.0 (1.0–1.0)	0.20 (0–0.44)	0.97 (0.93–0.99)
SAT%	0.54 (0.33–0.66)^a^	0.50 (0.47–0.54)^a^	0.50 (0.33–0.74)^a^	0.87 (0.80–0.92)^a^	0.48 (0.39–0.58)^a^

*Note*: ‘CLASS’ above denotes the algorithm in its entirety. This is compared to CLASS without ASR, with uniform segmentation of 1 s (USG 1) and 5 s (USG 5) (instead of ASG) and final classification using standard deviation alone (SD) (instead of multiple features and clustering).

a Denotes significant differences between values at each stage and CLASS in its entirety, at *p* < 0.05 using the paired *t*-test. IQR: interquartile range.

**Table 3:** Regression results for mean burst percentage (Burst%) and mean relative spectral power in delta, theta, and beta frequency bands. The log-transform of results are shown for CLASS QS estimates and visually labelled estimates from the clinician, as well as for non-state specific EEG epochs, for 31–38 week PMA range (optimal CLASS performance). For each measure, the slope (or *b*-coefficient, *b*), standard error (SE) of *b*, 95% confidence interval (CI), and *p* < 0.05 significance is presented. In case of quadratic correlations, coefficients *b*_1_ and *b*_2_ of the equation are provided (*y* = *a* + *b*_1_*x* + *b*_2_*x*^2^). The alpha band power showed no significant correlations, and was therefore omitted from this table.

	CLASS QS estimates	Visual QS estimates	Non-state specific EEG
Log Burst%	*b* = 0.045 SE: 0.004 95% CI: (0.036 to 0.053) linear correlation with PMA, *p* < 0.001	*b* = 0.045 SE: 0.005 95% CI: (0.035 to 0.055) linear correlation with PMA, *p* < 0.001	*b* = 0.035 SE: 0.006 95% CI: (0.023 to 0.047) linear correlation with PMA, *p* < 0.001
Log relative delta power	*b* = −0.014 SE: 0.004 95% CI: (−0.021 to −0.006) linear correlation with PMA, *p* < 0.01	*b* = −0.013 SE: 0.004 95% CI: (−0.022 to −0.004) linear correlation with PMA, *p* < 0.01	*b* = −0.010 SE: 0.005 95% CI: (−0.019 to −0.000) linear correlation with PMA, *p* < 0.05
Log relative theta power	*b*_1_ = −0.679 SE: 0.284 95% CI: (−1.253 to −0.104) *b*_2_ = 0.011 SE: 0.004 95% CI: (0.002 to 0.019) quadratic correlation with PMA, *p* < 0.05	*b*_1_ = −0.673 SE: −0.320 95% CI: (−1.334 to −0.012) *b*_2_ = 0.010 SE: 0.005 95% CI: (0.000 to 0.020) quadratic correlation with PMA, *p* < 0.05	*b*_1_ = −0.281 SE: 0.333 95% CI: (−0.956 to 0.392) *b*_2_ = 0.005 SE: 0.005 95% CI: (−0.005 to 0.014) *no significant* correlation with PMA, *p* = 0.35
Log relative beta power	*b*_1_ = 1.071 SE: 0.342 95% CI: (0.377 to 1.765) *b*_2_ = −0.016 SE: 0.005 95% CI: (−0.026 to −0.006) quadratic correlation with PMA, *p* < 0.05	*b*_1_ = 1.103 SE: 0.576 95% CI: (−0.060 to 2.267) *b*_2_ = −0.016 SE: 0.008 95% CI: (−0.033 to −0.000) quadratic correlation with PMA, *p* = 0.056	*b*_1_ = 0.389 SE: 0.507 95% CI: (−0.646 to 1.411) *b*_2_ = −0.007 SE: 0.007 95% CI: (−0.0203 to 0.007 no significant correlation with PMA, *p* = 0.428
